# Temporal Associations between Actigraphy-Measured Daytime Movement Behaviors and Nap Sleep in Early Childhood

**DOI:** 10.3390/ijerph192215308

**Published:** 2022-11-19

**Authors:** Christine W. St. Laurent, Jennifer F. Holmes, Rebecca M. C. Spencer

**Affiliations:** 1 Department of Psychological and Brain Science, University of Massachusetts Amherst, Amherst, MA 01003, USA; 2 Institute of Applied Life Sciences, University of Massachusetts Amherst, Amherst, MA 01003, USA

**Keywords:** physical activity, nap, sleep, early childhood, sedentary

## Abstract

The purpose of this micro-longitudinal study was to explore daily associations between daytime movement behaviors (sedentary time and physical activity) and nap sleep in young children. In 298 children (age = 51.0 ± 9.6 months, 43.6% female), wrist-based actigraphy (mean wear time = 10 days) assessed sedentary time, total physical activity, and provided an estimate of nap sleep duration and efficiency. Multilevel logistic and linear regression models were used to examine temporal within-person relations between wake behaviors and nap sleep, and adjusted for overnight sleep duration between days of interest, age, sex, and socioeconomic status. Movement behaviors were not related to the likelihood of next-day napping, but when children were less sedentary (OR = 0.96; *p* < 0.001) or more active (OR = 1.01; *p* = 0.001) in the morning, they were more likely to nap that same day. Movement behaviors were not associated with nap sleep duration or efficiency. Conversely, on days children napped, they were less sedentary (B = −2.09, *p* < 0.001) and more active (B = 25.8, *p* < 0.001) the following day. Though napping and movement behaviors had some reciprocal relations, effect sizes in the present study were small. Further studies should examine children with more diverse sleep health and from different childcare settings.

## 1. Introduction

Daytime sleep (i.e., napping) is a characteristic component of 24 h sleep in early childhood [1,2]. The current American Academy of Sleep Medicine guidelines recommend that preschool-aged children (i.e., 3 to 5 years) attain 10 to 13 h of sleep throughout a daily cycle, which may include nap sleep [3]. Sleep behaviors typically undergo a transition during the preschool years as children shift from biphasic (i.e., a daytime nap bout and an overnight sleep bout) to monophasic sleep (i.e., a single overnight sleep bout) [1]. Although most children cease napping by 5 years [2], the change in sleep consolidation may occur at different times among children [4].

Napping behaviors in children have been associated with a range of factors such as family routines, childcare or preschool attendance, parental sleep habits, technology use, and brain development [4,5]. These factors have also been influenced by daytime movement behaviors in young children (i.e., sedentary behaviors and physical activity) and therefore it is possible that such behaviors are also related to nap sleep [6,7]. Recent reviews on nap sleep and health factors have reported beneficial associations between daytime sleep and learning, school readiness, cognitive performance, behavior, and some metabolic health indicators [8,9]. However, it was noted that the role of nap sleep on physical health factors, such as movement behaviors, has not been sufficiently explored to draw informed conclusions.

Lower levels of sedentary behavior and higher levels of physical activity have been beneficially associated with sleep measures in adults, and there is some evidence that sleep can positively influence physical activity levels [10,11]. Although relations between movement behaviors and sleep in early childhood have been inconsistent, studies have been limited and most have solely examined overnight sleep—often not taking daytime sleep into account in analyses [12,13]. It is possible that napping could contribute to increased feelings of energy and motivation for movement, and high levels of movement could in turn improve the quantity and quality of nap sleep [14]. Among the few observational studies that have reported associations between nap sleep measures and movement behaviors in toddlers or preschool-aged children, findings have been generally null [15,16,17] or mixed [18]. However, these studies only examined between person associations with aggregated measures (e.g., using each participant’s average for the whole wear period of each of the behaviors in the statistical models).

Analyses of repeated measures can explore the temporal nature of relations between nap sleep and wake behaviors, as well as determine if there are within-person associations. Consideration of within-person relations can provide valuable information about how daily behaviors are influenced when a person does more or less of the exposure behavior of interest than their norm [19]. This type of approach can provide additional and translational information regarding the relations between sleeping and movement behaviors to what is traditionally reported from between-person reports [20,21]. Indeed, intensive longitudinal analyses have been used in studies of older children to investigate daily associations between sleep and physical activity [22,23,24,25,26,27,28]. In our recent report of temporal relations in preschool children, we found that while days with greater activity generally were not associated with sleep on the subsequent nights, nights with greater sleep efficiency appeared to beneficially influence the next day wake behaviors (i.e., less sedentary time and greater physical activity) [29]. To our knowledge, nap sleep and movement behaviors in early childhood have not yet been examined with this approach.

Therefore, the present study used an observational, micro-longitudinal design to explore temporal (day-to-day) associations between actigraphy-measured movement behaviors and nap sleep in preschool-aged children. Our first aim was to determine if children’s sedentary behavior or physical activity levels predicted their likelihood of napping the next day, and if less sedentary behavior and more activity predicted a greater likelihood to nap that same afternoon. Our second aim was to determine if, on days when children napped, their sedentary behavior or activity of the previous day predicted their nap duration or sleep efficiency, and if their movement behaviors of that morning predicted their nap duration or sleep efficiency that afternoon. Our third aim examined next day movement behaviors following a day that included a nap.

## 2. Materials and Methods

An intensive longitudinal analysis approach with observational data derived from a larger study (ClinicalTrials.gov ID: NCT09285880), conducted between 2013 and 2020, was used in this study. This report follows the Strengthening the Reporting of Observational Studies in Epidemiology (STROBE) checklist [30]. The University of Massachusetts Amherst Institutional Review Board approved the study protocol (approved 15 December 2011; protocol ID: 2011-1152). Adult caregivers provided informed consent for themselves and permission for their children’s participation. Verbal assent was obtained from the child participants. Childcare providers also provided informed consent.

### 2.1. Participants

**Figure 1:**
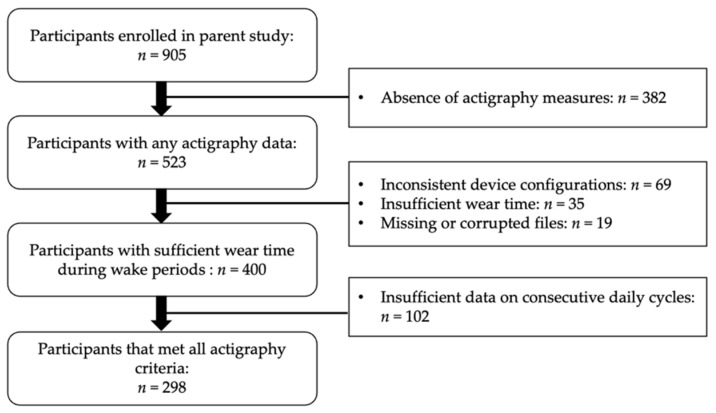
Participant flow diagram.

Participants included in this secondary data analysis attended preschool and childcare centers in western Massachusetts between 2013 and 2020 (Figure 1). Eligibility criteria to participate in the parent study included that the child: (1) was between 33 and 71 months of age, (2) had normal or corrected-to-normal vision and hearing, (3) had no current or previously diagnosed sleep disorder or developmental disability, (4) did not use psychotropic or sleep-affecting medications, and (5) did not travel outside of the local time zone within the previous week. This age range was selected as it represents the ages that most US children attend preschool educational and care programs. Additionally, with such centers, classrooms are usually separated by age group and preschool children are typically housed together. Additional inclusion criteria for the present study included at least three days with both daytime movement behavior and nap sleep actigraphy measures, with complete sleep and wake data for at least two consecutive daily cycles. 

### 2.2. Procedure

The aim of the parent study was to explore the effect of nap sleep on memory performance in US preschool-aged children, and therefore the protocol included two experimental conditions, both of which were completed by all participants approximately one week apart on a weekday with the order of conditions counterbalanced. The experimental conditions included nap promotion one afternoon and wake promotion on the other. Aside from these two days, participants followed their typical routines and schedules over the course of the study period (approximately 16 continuous days).

Upon enrollment, caregivers were given a health and demographics questionnaire to complete at any time throughout the study period and a sleep diary to report daily sleep information for their child. Children were asked to begin wearing an actigraphy monitor on the wrist of their non-dominant arm. They were asked to wear the watch for 24 h a day throughout the study period and were instructed to press an event marker on the watch to denote times in and out of bed. For days that children were at preschool, childcare providers were asked to complete a nap sleep log. Monetary compensation was provided to the caregivers and childcare providers and an age-appropriate book was given to the children at the completion of the study.

### 2.3. Measures

Actigraphy was collected with Spectrum Actiwatches to assess daily sedentary time, physical activity, and sleep measures (i.e., daytime nap and overnight sleep). Actigraphy data were processed with the Actiware software (Philips Respironics, Bend, OR, USA). The Actiwatch device uses a triaxial accelerometer, is water-resistant, provides off-wrist detection, and as mentioned above, is equipped with a button to mark events. In children, it has acceptable validity for sleep assessment relative to polysomnography [31,32]. Childhood sleep studies [31,33] have often used Actiware’s default algorithm [34]. Relative to videosomnography in children 28 to 73 months, a validity study reported 94% agreement, 97% sensitivity, and 24% specificity [35]. For waking behaviors in preadolescent children, the Actiwatch has been validated against indirect calorimetry for estimating energy expenditure (r = 0.90, *p* < 0.001) [36]. Activity count cut points were proposed from this study for thresholds of classifying wake behaviors into sedentary time and physical activity intensities. These cut points were cross-validated in a sample of preschool children relative to direct observation (r_sp_ = 0.47, *p* < 0.001) [37]. 

Prior to data collection, Actiwatches were configured in Actiware to collect data in 15-s epochs with a sampling rate of 32 Hz and a sensitivity of <0.01 g. When data collection concluded, actigraphy data were downloaded in Actiware and epochs were automatically scored as sleep, wake, or excluded (i.e., off-wrist). Actograms were then scored by trained research assistants using a combination of event markers, study diaries, and Actiware’s epoch classifications to define time intervals of wake and rest (time in bed) [34]. A daily cycle was defined by morning wake onset of one day until wake onset of the subsequent day.

When event markers and sleep diaries were not available, the start of the rest interval was defined as the first three consecutive minutes of sleep and the end of the rest interval was defined as the last five consecutive minutes of sleep. Actiware’s algorithm was then applied to the daytime and overnight rest intervals to determine sleep and wake epochs within those intervals. In our sample, none of the participants had more than one distinct overnight rest interval. Epochs that were classified as sleep during rest intervals contributed to sleep time and epochs classified as wake during a rest interval contributed to wake time in bed. Variables for each nap and overnight rest interval included time in bed (min), sleep duration (time in bed classified as sleep; min), and sleep efficiency (sleep duration divided by time in bed multiplied by 100%). Weekly nap frequency was also calculated (i.e., the number naps/number of non-experimental days multiplied by seven).

Physical activity and sedentary time were calculated by further processing of the daytime wake intervals. Movement behaviors were only computed with days that included at least 480 min of wear time [38]. Activity counts during daytime wake intervals were summed for each day. Overall physical activity was parameterized as the average daily activity counts/min. Sedentary time was calculated as the sum of epochs with activity counts below 79, as defined by Ekblom et al. [36], and presented as percent of wake time during the day. Physical activity and sedentary time variables were also created for morning periods only (i.e., non-time in bed wake periods between 12 a.m. and 12 p.m.).

A health and demographics questionnaire was completed by adult caregivers to provide information about the children’s age, sex, socioeconomic status, race, and ethnicity. Reported household income, parent employment status, and highest level of education were used to calculate a composite socioeconomic score (range 0 to 7, with higher scores reflecting higher status) [39].

### 2.4. Analysis

Statistical analyses were conducted in Stata (Version 17.0, StataCorp LLC, College Station, TX, USA) with an alpha level of 0.05. Multilevel models with lagged effects with an autoregressive [1] error covariance structure (for linear models) were used to examine temporal relations between daytime movement behaviors and nap sleep measures. Independent variables were centered around the means. As our research questions focused on within-person differences, variables were centered around individuals’ means. Two models were run for each of our research questions—one with sedentary time as the exposure of interest and one with physical activity as the exposure of interest. Model assumptions (e.g., constant variance and linearity for linear models) were met.

Our first aim was to explore if there were temporal associations between (1) movement behaviors and the likelihood of napping the next day and (2) morning movement behaviors and the likelihood of napping that same day. Multilevel logistic regression models were used for these analyses with nap occurrence as the binary outcome variable (i.e., yes or no). To explore the occurrence of napping on the next day (n = 2 models), time varying predictors included the previous days within-person sedentary time or physical activity and night sleep duration (i.e., of the night between the two days), and time invariance predictors included, age, sex, and socioeconomic score. To explore likely occurrence of napping on the same day (n = 2 models), time varying predictors included that same morning’s within-person sedentary time or physical activity and the previous night’s sleep duration, as well as the same time invariant variable included above. 

Our second focus was to examine if when children did nap, (1) were movement behaviors of the previous day associated with the nap duration or sleep efficiency and (2) did movement behaviors of that same morning predict their nap duration or sleep efficiency. These aims were addressed with multilevel linear models with nap duration or nap sleep efficiency as the outcomes. The first two models included the previous day’s within-person sedentary time or physical activity and night sleep duration (i.e., the night between the two days) as time varying predictors, and age, sex, and socioeconomic status score as time invariant predictors. The second two models included the same day’s within-person morning sedentary time or physical activity and previous night’s sleep duration as time varying predictors, and the same aforementioned time invariant predictors.

Our final question was to explore whether children were less sedentary and more active on the day following a nap. Again, multilevel linear regression models (n = 2) were used, this time with sedentary time or physical activity as the outcomes. Time varying predictors were the previous day’s within-person nap status (i.e., yes or no) and night sleep duration (i.e., the night between the two days) and the time invariant covariates were again age, sex, and socioeconomic status.

## 3. Results

### 3.1. Participant Characteristics

The descriptive characteristics of our final sample (n = 298) are presented in Table 1. Movement behaviors were measured for 3 to 15 days (M = 10 ± 3.6 days) including 7.9 ± 2.5 weekdays and 2.5 ± 1.4 weekend days with an average of 724.22 min of daily wear time during wake. Data were collected at various time points at each school, with approximately an even distribution of participants measured across months and seasons. Although children on average napped on about half of the days of the week, the nap frequency of this sample ranged from 0 to 7 days. Children participated in slightly less sedentary time and more physical activity on mornings relative to the full day. Most children met overall guidelines for 24-h sleep duration [3]. However, sleep efficiency was lower for nap bouts (85.2%) relative to overnight sleep (88.3%).

### 3.2. Likelihood of Napping

Odds ratios and confidence intervals for the multilevel logistic regression models exploring movement behaviors on the likelihood of napping are presented in Table 2. Participating in sedentary (*p* = 0.823) or physical activity (*p* = 0.315) levels that differ from a typical day did not increase the likelihood of taking a nap the following day. Morning movement behaviors were associated with the likelihood of napping that same day, albeit with very small effect sizes. When children participated in more sedentary time than their typical morning, they were 0.04 times less likely to nap that afternoon (*p* < 0.001). When children were more active relative to their average morning levels, they were 0.01 times more likely to nap (*p* = 0.001).

### 3.3. Nap Duration and Sleep Efficiency

Regression coefficients and confidence intervals for the multilevel linear regression models exploring the associations of movement behaviors on nap sleep duration and efficiency are presented in Table 3. When children did nap, their levels of sedentary behavior (*p* = 0.173) and physical activity (*p* = 0.132) of the previous day were not associated with their next day nap duration. Likewise, the previous day’s sedentary behavior (*p* = 0.723) and physical activity (*p* = 0.618) were not associated with nap sleep efficiency. Null associations were also observed when exploring relations between morning movement behaviors and nap sleep measures. Neither sedentary time nor physical activity in the morning were associated with nap duration (*p* = 0.380 and *p* = 0.143, respectively) or sleep efficiency (*p* = 0.650 and *p* = 0.312, respectively).

### 3.4. Next Day Movement Behaviors

Regression coefficients and confidence intervals the multilevel linear regression models exploring the association between the occurrence of a nap and next day movement behaviors are presented in Table 4. When children did take a nap, they were more like to be less sedentary (*p* < 0.001) and more active (*p* < 0.001) the following day, yet again with relatively small effect sizes. Specifically, taking a nap was associated with a 2.1% decrease in time spent sedentary and an increase of 25.8 activity counts/min.

## 4. Discussion

Previous studies exploring relations between sleep and movement behaviors in early childhood have primarily focused on overnight sleep and between-person associations. Relations between nap sleep and such behaviors is unclear and understanding how individual variations of these factors may influence each another can be helpful to parents and caregivers. Therefore, the aim of the present study was to explore daily associations between daytime movement behaviors and nap sleep in young children. In this sample of children that attended preschool or childcare centers, movement behaviors of the previous day did not influence the likelihood of napping, but morning behaviors did influence nap likelihood that same day. There were no associations between movement behaviors of the previous day or same morning on nap sleep metrics. Finally, taking a nap was associated with less sedentary time and more activity on the next day. However, when interpreting these findings, it should be considered that the significant associations observed here had small effect sizes.

Suggested physiological and behavioral pathways between movement behaviors and sleep stem from studies in adults [10,11]. Research is needed to determine what mechanisms may play a role in children [13]. However, in respect to why a child’s movement behaviors of the previous day would influence the likelihood of them napping, some of the supported explanations in adults may translate to young children. For example, engaging in higher levels of physical activity translates to greater energy expenditure which, in turn, could contribute to greater physical fatigue that may induce more sleep pressure [10]. Moreover, activities of preschool children are often cognitively engaging (e.g., integrating motor skills and changing directions and speed—rather than just continuous and repetitive activities [40,41,42]) and this may also contribute to increased sleep pressure and need [5].

The present findings did not provide support that movement behaviors of either the previous day or same morning are influential on nap sleep duration or efficiency. These null associations appear to be comparable to studies that have examined between-person nap and movement behavior relations in young children [15,16,17]. In our recent study exploring daily associations, night sleep efficiency, at the within-person level, was associated with next day’s movement behaviors, but these relations were not bidirectional (i.e., movement behaviors did not predict that night’s sleep efficiency) [29]. The same sample was used for this previous report and the present study, and most children still obtained a recommended amount of sleep even on days that were shorter relative to their typical nights. Therefore, exploring effects of movement behaviors on nap metrics in a more diverse sample with more diverse sleep health (e.g., children with more variable sleep patterns) may be an informative next step.

The decrease in sedentary time and increase in physical activity following a day with a nap may be most meaningful finding of the current study for caregivers. Studies in adults have highlighted some aspects relating to movement and performance that are benefitted by daytime napping. For example, among physically active adults, recovery and performance metrics of activity (e.g., power, reaction time, and endurance) appear to benefit from a daily nap [14]. These pathways have yet to be fully explored, but could potentially exist in young children. Indeed, in Chinese primary students, daytime napping has been shown to be positively associated with various behavioral health, psychological, and cognitive measures [9], many of which are also factors that correlate with preferred movement behavior profiles [6].

While the current study included objective measures of sleep and wake and used a unique approach of the relations in this population, some considerations should be taken into account. First, additional contexts of movement behaviors (e.g., types of activity and indoor versus outdoor settings) and nap sleep (e.g., sleep environment, location—preschool or home, and reason for nap) may be helpful to better understand these relations. Second, given that this sample of children attended childcare or preschool centers at least some days of the week, their behaviors may have been restrained around routines (e.g., facility space and schedule may limit movement opportunities, or the nap may be required or optional), which may limit the generalizability. Additionally, generalizability should also be limited young children within the same age group, particularly as infants and toddlers have different sleep and wake patterns than preschool-aged children [1,43]. Finally, as both wake and sleep routines may vary on different types of days (e.g., weekday versus weekend days and days a child attends childcare versus stays at home), future research could consider potential modifying factors of the relations between nap sleep and movement behaviors.

## 5. Conclusions

Although we found most associations between nap sleep and movement behaviors in preschool children to be null, using a within-person approach with an analysis that explores daily (rather than aggregated) behaviors did highlight some novel relations that have not been observed in previous between-person studies. As napping in early childhood is beneficial towards learning and memory development [44,45], our finding that children’s waking behaviors influence their likelihood of napping the next day is particularly informative. Additionally, this suggests another clear importance of early childhood physical activity. Our preliminary findings can be used to inform future sleep and physical activity interventions. However, additional research to explore behavior contexts in healthy diverse populations would be a suggested next step.

## Figures and Tables

**Table 1:** Descriptive characteristics of the study sample (n = 298).

Variables		Mean (SD) or n (%)	Range
**General characteristics**			
Age (months)		51.0 (9.6)	33 to 70
Sex (female)		130 (43.6)	-
Socioeconomic status (score: 0 to 7)		4.5 (2.0)	2 to 7
Race			
	White	183 (61.3)	-
	Black/African American	26 (8.7)	-
	Asian	13 (4.4)	-
	Native Hawaiian/Pacific Islander	2 (0.7)	-
	Two or more racial groups	34 (11.4)	-
	Other	21 (7.1)	-
	Missing	19 (6.4)	-
Hispanic			
	Yes	79 (69.5)	-
	No	207 (26.5)	-
	Missing	12 (4.0)	-
**Nap sleep**			
	Duration (min) *	91.7 (25.9)	24.6 to 221
	Efficiency (%)	85.2 (10.6)	37.5 to 98
	Frequency (#/week)	3.7 (2.0)	0 to 7
**Daytime movement behaviors**			
	Sedentary time (%)	41.8 (7.6)	18.3 to 65.4
	Total physical activity (counts/min)	565.73 (104.24)	278.69 to 1025.68
**Morning movement behaviors** **			
	Sedentary time (%)	39.6 (8.6)	14.3 to 70.0
	Total physical activity (counts/min)	579.59 (115.26)	247.29 to 1049.63
**Overnight sleep**			
	Duration (min)	570.31 (40.1)	4660.15 to 690.97
	Efficiency (%)	88.3 (3.4)	78.5 to 95.5

* Determined only on days children napped; ** Wake out of bed between 12 a.m. and 12 p.m.

**Table 2:** Multilevel logistic regression model results for likelihood of napping.

	Next Day Nap		Same Day Nap	
	OR (SE)	95% CI	OR (SE)	95% CI
**Models with sedentary time**				
Sedentary time	1.00 (0.009)	0.98 to 1.0	0.96 (0.009)	0.94 to 0.98
Night sleep	0.99 (0.001)	0.99 to 1.0	0.99 (0.002)	0.99 to 1.0
Day	0.94 (0.02)	0.91 to 0.98	0.93 (0.02)	0.90 to 0.97
Age	0.94 (0.01)	0.92 to 0.97	0.94 (0.01)	0.92 to 0.97
SES	0.93 (0.05)	0.84 to 1.0	0.89 (0.05)	0.79 to 0.99
Sex	0.90 (0.19)	0.59 to 1.4	0.92 (0.22)	0.58 to 1.5
Intercept	1.47 (0.22)	-	1.67	-
**Models with physical activity**				
Physical activity	0.99 (0.0008)	0.99 to 1.00	10.01 (0.0007)	1.001 to 1.004
Night sleep	0.99 (0.001)	0.99 to 1.0	0.99 (0.001)	0.99 to 1.00
Day	0.94 (0.02)	0.91 to 0.98	0.93 (0.02)	0.90 to 0.97
Age	0.95 (0.01)	0.93 to 0.97	0.95 (0.01)	0.92 to 0.97
SES	0.93 (0.05)	0.84 to 1.1	0.89 (0.05)	0.79 to 1.0
Sex	0.90 (0.19)	0.60 to 1.4	0.98 (0.22)	0.63 to 1.52
Intercept	1.49 (0.23)	-	1.6 (0.27)	-

OR = odds ratio; SE = standard error; CI = confidence interval; SES = socioeconomic status.

**Table 3:** Multilevel linear regression results for nap sleep measures.

	Nap Duration		Nap Sleep Efficiency	
	Coef. (SE)	95% CI	Coef. (SE)	95% CI
**Models with sedentary time**				
Model 1				
Previous day’s sedentary time	0.29 (0.22)	−0.12 to 0.72	0.02 (0.08)	−0.13 to 0.19
Previous night’s sleep	0.01 (0.03)	−0.04 to 0.07	−0.002 (0.01)	−0.03 to 0.02
Day	0.17 (0.37)	−0.55 to 0.89	0.02 (0.15)	−0.28 to 0.32
Age	−0.42 (0.19)	−0.70 to −0.05	−0.21 (0.09)	−0.38 to −0.04
SES	−1.7 (0.93)	−3.4 to 0.15	−0.48 (0.43)	−1.3 to 0.36
Sex	−1.8 (3.6)	−8.9 to 5.2	−0.10 (1.7)	−3.4 to 3.2
Intercept	93.4 (2.6)	-	85.5 (1.2)	-
Model 2				
Morning sedentary time	0.17 (0.19)	−0.20 to 0.53	0.03 (0.07)	−0.11 to 0.17
Previous night’s sleep	0.0007 (0.03)	−0.05 to 0.06	−0.002 (0.012)	−0.03 to 0.02
Day	0.42 (0.34)	−0.24 to 1.1	0.07 (0.14)	−0.21 to 0.35
Age	−0.46 (0.18)	−0.81 to −0.12	−0.19 (0.09)	−0.36 to −0.01
SES	−1.6 (.86)	−3.3 to 0.07	0.54 (0.44)	−1.4 to 0.31
Sex	−1.3 (3.3)	−7.9 to 5.2	−0.26 (1.7)	−3.6 to 3.1
Intercept	92.6 (2.4)	-	85.7 (1.2)	-
**Models with physical activity**				
Model 1				
Previous day’s physical activity	−0.02 (0.16)	−0.05 to 0.007	−0.003 (0.007)	−0.02 to 0.01
Previous night’s sleep	0.02 (0.03)	−0.04 to 0.07	−0.004 (0.01)	−0.03 to 0.02
Day	0.19 (0.37)	−0.53 to 0.91	0.04 (0.15)	−0.26 to 0.02
Age	−0.42 (0.19)	−0.79 to −0.05	−0.21 (0.09)	−0.38 to −0.04
SES	−1.7 (0.93)	−3.5 to 0.11	−0.46 (.43)	−1.3 to 0.38
Sex	−1.5 (3.6)	−8.6 to 5.6	−0.13 (1.7)	−3.4 to 3.2
Intercept	93.1 (2.6)	-	85.5 (1.2)	-
Model 2				
Morning physical activity	−0.02 (0.01)	−0.05 to 0.007	−0.006 (0.006)	−0.02 to 0.006
Previous night’s sleep	0.004 (0.03)	−0.05 to 0.06	−0.002 (0.01)	−0.03 to 0.02
Day	0.36 (0.34)	−0.31 to 1.0	0.08 (0.14)	−0.19 to 0.36
Age	−0.48 (0.18)	−0.82 to −0.13	−0.18 (0.09)	−0.36 to −0.01
SES	−1.7 (0.87)	−3.4 to −0.01	−0.49 (0.44)	−0.13 to 0.37
Sex	−1.9 (3.4)	−8.6 to 4.7	−0.68 (1.7)	−4.1 to 2.7
Intercept	93.2 (2.5)	-	85.9 (1.3)	-

Coef = regression coefficients; SE = standard error; CI = confidence interval; SES = socioeconomic status.

**Table 4:** Multilevel linear regression results for movement behaviors.

	Sedentary Time		Physical Activity	
	Coef (SE)	95% CI	Coef (SE)	95% CI
Previous day nap occurrence	−2.09 (0.57)	−3.2 to −0.99	25.8 (6.9)	12.3 to 39.3
Previous night’s sleep	−0.006 (0.005)	−0.02 to 0.003	0.10 (0.06)	−0.006 to 0.22
Day	0.08 (0.06)	−0.04 to 0.21	−0.37 (0.76)	−1.9 to 1.1
Age	−0.12 (0.06)	−0.23 to −0.02	2.5 (0.72)	1.1 to 3.9
SES	−0.46 (0.27)	−0.98 to 0.07	1.1 (3.5)	−5.7 to 7.9
Sex	−0.96 (1.1)	−3.0 to 1.1	45.9 (13.8)	18.8 to 73.0
Intercept	42.7 (0.76)	-	539.76 (9.86)	-

Coef = regression coefficients; SE = standard error; CI = confidence interval; SES = socioeconomic status.

## Data Availability

The data presented in this study are available on request from the corresponding author.
